# Recovery of left ventricular systolic function in peripartum cardiomyopathy: an observational study from rural Tanzania

**DOI:** 10.1186/s12872-024-03906-y

**Published:** 2024-05-09

**Authors:** Andrew Katende, Laurine Roos, Victor Z. Urio, Evance Mahundi, Victor Myovela, Dorcas Mnzava, Chipegwa Mlula, Christamonica Chitimbwa, Dominick M. Raphael, Winfrid Gingo, Fabian C. Franzeck, Daniel H. Paris, Luigia Elzi, Maja Weisser, Martin Rohacek

**Affiliations:** 1 St. Francis Regional Referral Hospital, Ifakara, United Republic of Tanzania; 2 Ifakara Health Institute, Ifakara, United Republic of Tanzania; 3 Swiss Tropical and Public Health Institute, Allschwil, Switzerland; 4 University of Basel, Basel, Switzerland; 5 Research and analytics services, University Hospital Basel, Basel, Switzerland; 6 Regional Hospital of Bellinzona e Valli, Bellinzona, Switzerland; 7 Division of Infectious Diseases and Hospital Epidemiology, University Hospital Basel, Basel, Switzerland; 8 Swiss Tropical and Public Health Institute (Swiss TPH), Kreuzstrasse 2, Allschwil, 4123 Switzerland

**Keywords:** Peripartum cardiomyopathy, Recovery, Left Ventricular Systolic Function, Rural, Tanzania, Sub-Saharan Africa

## Abstract

**Background:**

The aim of this study was to evaluate the recovery rate of the left ventricular systolic function of women diagnosed with peripartum cardiomyopathy receiving specialized care in rural Tanzania.

**Methods:**

In this observational study, women diagnosed with peripartum cardiomyopathy at a referral center in rural Tanzania between December 2015 and September 2021 were included. Women diagnosed between February and September 2021 were followed prospectively, those diagnosed between December 2015 and January 2021 were tracked back for a follow-up echocardiography. All participants received a clinical examination, a comprehensive echocardiogram, and a prescription of guideline-directed medical therapy. The primary outcome was recovery of the left ventricular systolic function (left ventricular ejection fraction > 50%).

**Results:**

Median age of the 110 participants was 28.5 years (range 17–45). At enrolment, 49 (45%) participants were already on cardiac medication, 50 (45%) had severe eccentric hypertrophy of the left ventricle, and the median left ventricular ejection fraction was 30% (range 15–46). After a median follow-up of 8.98 months (IQR 5.72–29.37), 61 (55%) participants were still on cardiac medication. Full recovery of the left ventricular systolic function was diagnosed in 76 (69%, 95% CI 59.6–77.6%) participants. In the multivariate analysis, a higher left ventricular ejection fraction at baseline was positively associated with full recovery (each 5% increase; OR 1.7, 95% CI 1.10–2.62, *p* = 0.012), while higher age was inversely associated (each 10 years increase; OR 0.40, 95% CI 0.19–0.82, *p* = 0.012).

**Conclusion:**

Left ventricular systolic function recovered completely in 69% of study participants with peripartum cardiomyopathy from rural Tanzania under specialized care.

**Supplementary Information:**

The online version contains supplementary material available at 10.1186/s12872-024-03906-y.

## Introduction

Peripartum cardiomyopathy (PPCM) is a potentially life-threatening condition typically presenting as heart failure with reduced ejection fraction in the last month of pregnancy or within 5 months following delivery in women without any known cause of heart failure [1, 2].

In the United States of America (USA), PPCM affects 1 in 1000 to 4000 pregnant women with the lowest incidence in Hispanics and the highest in African Americans. In South Africa, 1 in 1000 deliveries is affected, in Haiti 1 in 300 and in North-western Nigeria 1 in 100 [3]. At the Saint Francis Regional Referral Hospital in rural Tanzania, in a series of 815 adults diagnosed with a heart disease within 23 months, PPCM was diagnosed in 11.5% of female study participants with a median left ventricular ejection fraction (LVEF) of 30% [4].

This variable geographical occurrence of PPCM indicates the influence of environmental risk factors and genetic susceptibility to PPCM. The most relevant known risk factors for PPCM are ethnic background, preeclampsia, smoking, multiparity, and multifetal pregnancy [5]. Hypothesized pathophysiological pathways include inflammatory and autoimmune processes, and enhanced oxidative stress leading to increased cardiac cathepsin D expression and activity which results in the formation of a cleaved antiangiogenic and proapoptotic 16 kilo Dalton form of the hormone prolactin leading to vascular and myocardial dysfunction [3, 5]. The full recovery rate of the left ventricular (LV) systolic function has been reported to be 21% in South Africa after 6 months, 24% in Nigeria after 17 months, 71% in the USA after 1 year, and 72% in Germany after 5 years [6–9]. All-cause maternal mortality at 6 months ranged from 2.9% in the USA to 10.9% in Africa [10], and neonatal death occurred in 5% in a large global registry [11]. However, in contrast to participants from the USA and Europe who were diagnosed early and received specialized care, participants from Nigeria were diagnosed late and were not optimally treated. Participants included in these studies were living in urban areas. Inhabitants of rural areas might experience poorer health services, poorer access to medication, and experience lower life expectancy [12]. To date, information about characteristics, prescribed medication, and outcomes of women with peripartum cardiomyopathy and newborns living in rural areas of sub-Saharan Africa are scarce.

The aim of this study was to determine the proportion of women with recovery of the LV systolic function among participants with PPCM from rural Tanzania who received specialized care, to evaluate clinical and echocardiographic characteristics and adherence to recommended medical therapy, to determine factors associated with LV systolic function recovery, and to describe outcomes of the newborn.

## Materials and methods

### Study design and study site

This was an observational single-center study of women diagnosed with PPCM at the Saint Francis Regional Referral Hospital in Ifakara, Tanzania. This is a referral hospital for a rural population of about 1 million inhabitants from the rural Kilombero-, Ulanga- and Malinyi districts, living up to 160 km away from the hospital. We prospectively recruited women with PPCM diagnosed at this center from February 1st 2021 to September 30st 2021, and followed them for a minimum of 6 months. In addition, women who had been diagnosed with PPCM between December 1st 2015 and January 31st 2021 were called back for a follow-up examination at the heart and lung clinic of the hospital which is operating since May 2021. This clinic provides specialized care for patients with chronic heart- and lung diseases and is run by physicians and trained clinical officers. Before 2021, patients with heart failure were diagnosed and followed by an experienced physician (MR) skilled in echocardiography. All participants were seen by a physician or clinical officer at baseline and during follow-up, and received a prescription of guideline directed medical therapy for heart failure. Therapy was titrated upward, and participants were carefully counselled according to the recommendations of the European Society of Cardiology [1, 13].

### Study population

Eligible were all women who received an echocardiogram at the hospital and were diagnosed with peripartum cardiomyopathy according to the definition of the European Society of Cardiology (i.e. cardiomyopathy with a LVEF < 45%, presenting towards the end of the pregnancy, or within 5 months after delivery in a woman without previously known structural heart disease) [1, 2]. The baseline echocardiogram was used to diagnose PPCM. Women with pre-existing cardiac disease were excluded from the study. All included study participants signed an informed consent form. The child of the affected pregnancy was included after the mother signed the informed consent form.

### Data collection

Women diagnosed with PPCM presenting between February 1st 2021 to September 30st 2021 were prospectively enrolled, consented and scheduled for a visit after 6 months or earlier if clinically indicated. Clinical and echocardiographic characteristics of women diagnosed with PPCM between December 1st 2015 and January 31st 2021 were retrieved from the routine electronic database (EpiData®). These data were accessed on October 31st 2020 before finishing the study protocol and January 31st 2021 before starting the prospective enrolment period. Patients were traced by phone call, radio announcement or tracked by healthcare workers and invited to participate in this study. Investigators who examined participants had access to data that could be used to identify individual participants, but data were anonymized before they were sent to the statistician for analysis.

At enrolment, a systematic questionnaire was used to collect information on symptoms, co-morbidities, obstetric history, outcome of the newborn, and adherence to medication. A detailed clinical examination including signs and symptoms of heart failure was performed. Blood pressure was measured with an Omron M2 basic blood pressure device in sitting position after at least 5 min of rest. Arterial hypertension was defined as a blood pressure of ≥140/90mmHg [14].

Electrocardiograms (ECG) were performed at baseline and follow-up on the prospectively enrolled participants, and at follow-up on the retrospectively enrolled participants using a Schiller Cardiovit MS-2015 machine. Comprehensive echocardiograms at baseline and follow-up were done using a Mindray M7 or Chison Sonobook 9 ultrasound machine including a phase array sector probe according to current recommendations [15–18]. Echocardiographic parameters including LVEF, left and right atrial and ventricular dimensions, diastolic function, right ventricular function, and valvular abnormalities were documented. All left ventricular measurements to calculate the LVEF were measured using the biplane Simpson method of discs [15]. The diameters of left ventricles were measured in B-mode. Left atria were measured biplane in the four-chamber and two-chamber views. Diastolic function was assessed according to the recommendations of Nagueh et al. [16]. To calculate the LV mass, the LV mass index, the relative wall thickness (RWT), and the severity of mitral valve regurgitation, an online calculator provided by the Canadian Society of Echocardiography (https://csecho.ca/mdmath/) was used. Echocardiograms were performed by 3 experienced echocardiographers. All echocardiograms were reviewed by a second echocardiographer. In case of discrepancies regarding echocardiographic measurements, a third echocardiographer was involved to conclude on the correct measurements together. All ECGs were interpreted and reviewed by experienced physicians.

Laboratory tests were done at baseline for prospectively enrolled participants and at follow-up for those enrolled retrospectively. Laboratory data included brain natriuretic peptide (BNP), glucose, creatinine, and haemoglobin done in whole blood using the iSTAT® point of care system (Abbott, Chicago, USA).

Each study participant received health education about their medical condition. Furosemide, Enalapril, Carvedilol, and, if LVEF was below 35%, Spironolactone was prescribed. Data on birth weight, length and Apgar score of the newborn were collected from the antenatal care card.

At the follow-up visit, the history of symptoms and medication was assessed, a clinical examination performed, the echocardiography repeated, and a laboratory investigation was done, and the dose the angiotensin converting enzyme (ACE)-inhibitor and betablocker was increased. Participants not presenting to a planned follow-up visit were traced by healthcare workers or tracked physically. All data were entered into the electronic database using EpiData® (version 2.1) and stored on a local, password-protected laptop.

### Statistical analysis

Basic sociodemographic, clinical, laboratory and echocardiographic characteristics were compared between those with full recovery and those without using X^2^-test for categorical variables and Mann-Whitney U test for continuous variables. 95% confidence intervals for proportions were calculated according to the exact binominal method. Univariate and multivariate logistic regression were used to investigate predictors of LV recovery. All values were expressed with odds ratio (OR) and 95% confidence intervals (CI). A p-value of < 0.05 and a 95% CI not containing 1 were considered of statistical significance. At follow-up visit, reduced LVEF was defined as LVEF ≤ 50% and categorised into mildly- (LVEF 41–50%), moderately- (LVEF of 30–40%) and severely reduced LVEF (LVEF < 30%).

Full recovery of the LV systolic function was defined as LVEF > 50% after 6 months, an increase in the LVEF to 35–50% was defined as a partial recovery of the LV systolic function, and a non-recovered LV systolic function was defined as a LVEF of < 35% after at least 6 months. All statistical analysis were done using STATA version 13 for Windows.

## Results

### Enrolment

**Fig. 1:**
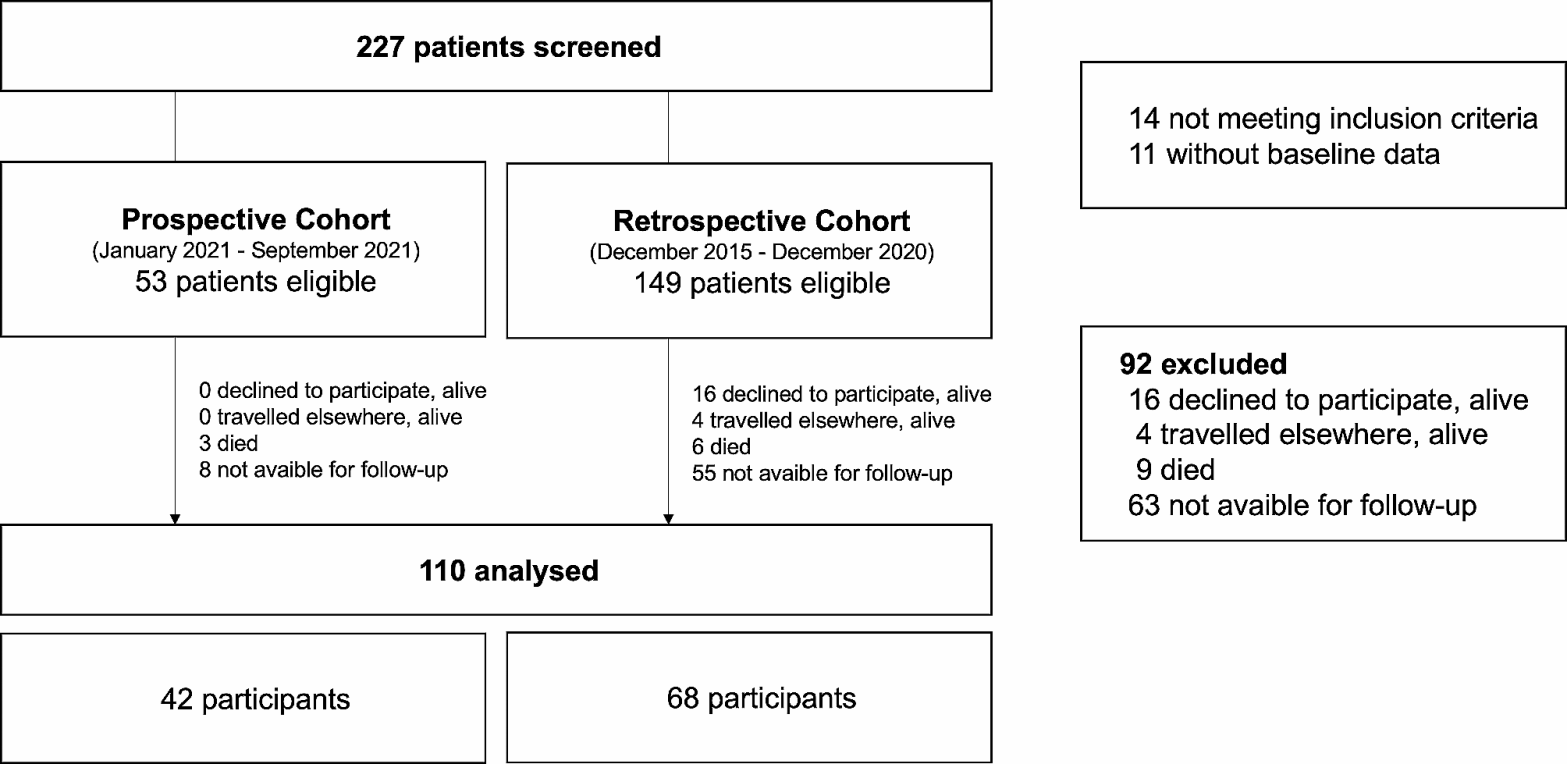
Enrolment of study participants

During the study period, 227 women were screened, and 202 were eligible (Fig. 1). We excluded 92 women. Of these 63 had to be excluded due to unavailability for a follow-up examination. 16 women declined to participate, 4 travelled elsewhere and 9 died. Overall, 110 participants were included in the final analysis (42 in the prospective cohort and 68 in the retrospective cohort). For all participants, median days between delivery and inclusion at baseline was 89 days (IQR 36–171).

### Baseline characteristics

The median age of the 110 included participants was 28.5 years (range 17–45). The median number of gravidities was 3 (range 1–8), and the median number of parities was 3 (range 1–7). A total of 12 (11%) participants had a multifetal pregnancy, 3 (3%) had a history of eclampsia, 2 (2%) were HIV-positive, 9 (8%) were obese (i.e., body mass index of 30 kg/m^2^ or above), 29 (26%) were hypertensive, 1 (1%) had diabetes (i.e., random blood glucose > 200 mg/dl), and 35 (32%) were admitted to the ward.

Before enrolment, 49 (45%) participants were on a cardiac medication due to clinical signs and symptoms of heart failure in the peripartum time period (42 participants on furosemide, 14 on ACE-inhibitor, 10 on a beta-blocking agent, 4 on spironolactone, 4 on digoxin, and 5 on bromocriptine). None of the participants had a history of previous cardiac disease.

At baseline, the median LVEF was 30% (range 15–46). A total of 60 (54%) participants had a moderately impaired and 37 (34%) a severely impaired left ventricular systolic function. In echocardiography, 50 (45%) participants had severe eccentric hypertrophy of the left ventricle. Table 1 shows participant’s baseline characteristics according to recovery of the LV systolic function: In the group with a later full recovery of the LV systolic function, participants were younger (median age 28 vs. 32 years, *p* = 0.040), had less orthopnoea (59.2% vs. 81.8%, *p* = 0.023), and had a higher LVEF at baseline (median LVEF 34% vs. 29%, *p* = 0.001). Table S1 shows detailed echocardiographic parameters of all included participants.

Among the 42 participants prospectively enrolled from February to September 2021, the group with full recovery of the LV systolic function exhibited more breastfeeding (96.7% vs. 58.3%, *p* = 0.001), reported less fatigue (36.7% vs. 83.3%, *p* = 0.006), and less palpitations (66.7% vs. 91.7%, *p* = 0.096), and had fewer repolarization abnormalities in the ECG at baseline (50% vs. 83.3%, *p* = 0.048). Haemoglobin, creatinine, blood glucose, and BNP were broadly similar in both groups (Table S2).

To ensure comparability of women with and without follow-up examinations, we compared baseline characteristics of excluded and included participants. Within the 92 excluded women, there were more inpatients (46.2% vs. 31.8%, *p* = 0.046), more with elevated blood pressure (36.9% vs. 21.8%, *p* = 0.018), and the LV mass index was larger (median 130 g/m^2^ (range 47–248) vs. 118 g/m^2^ (range 11–246), *p* = 0.044) than within the 110 participants who were included in the analysis. Age, profession (i.e. farmer), median LVEF (30% (range 15–50) vs. 30% (range 15–46), *p* = 0.091), the left ventricular end-diastolic diameter (median 57.5 mm (range 44–74 mm) vs. 57.1 mm (range 39–79 mm), *p* = 0.915), the left atrial volume index, and the frequency of severe LV eccentric hypertrophy and diastolic relaxation impairment grade 3 was broadly similar in both groups (Table S3).

**Table 1:** General characteristics of study participants (*n* = 110) at baseline according to recovery from peripartum cardiomyopathy

Variable	Full recovery (LVEF > 50%) (*n* = 76)		Partial or no recovery (*n* = 34)		*p*-value
	*n*	%	*n*	%	
**Demographic parameters**					
Age, median, IQR	28	24-33.5	32	25–39	**0.040**
BMI, median, IQR	22	20–25	24	22–27	0.159
Higher education (> primary school)	18	25.0	6	18.2	0.440
Farmer	63	82.9	27	79.4	0.662
Tribe: Msukuma	8	12.3	2	6.1	0.135
Mpogoro	15	23.1	12	36.4	
Mdamba	6	9.2	5	15.2	
Mbena	4	6.2	4	12.1	
Mngindo	4	6.2	4	12.1	
Mhehe	6	6.2	0	-	
other	22	33.9	6	18.2	
Inpatient at baseline	24	31.6	11	32.4	0.936
**Clinical parameters**					
Arterial hypertension	16	21.1	13	38.2	0.059
Intake of any cardiac medication	31	44.3	18	58.1	0.201
Chest pain	21	27.6	11	32.4	0.614
Dyspnoea (severity not specified)	60	79.0	30	88.2	0.243
Orthopnoea	42	59.2	27	81.8	**0.023**
**Echocardiographic parameters**					
LVEF, median, IQR	34	28–40	29	20–31	**0.001**
LVEDD, median, IQR	57	54–60	59	56–63	0.129
LVIDS, median, IQR	50	46–53	51	48–55	0.112
LV mass, median, IQR	196	164–217	184	170–218	0.900
LV mass index, median, IQR	118	101–143	118	106–136	0.883
LAVI, median, IQR	40	32–52	44	35–58	0.505
Diastolic relaxation impairment grade 3	44	59.4	26	78.8	0.052
Pulmonary arterial pressure, median, IQR	38	31–46	42	35–47	0.188
Mitral valve regurgitation, moderate or severe	4	5.3	2	5.9	0.605
Pericardial effusion	14	19.7	9	29.0	0.301
Apical thrombus	2	2.6	2	5.9	0.400
Elevated LV filling pressure	54	72.9	29	87.9	0.088

IQR, interquartile range; LVEF, left ventricular ejection fraction; LVEDD, left ventricular enddiastolic diameter; LVIDS, left ventricular internal diameter end systole; LV, left ventricular; LAVI, left atrial volume index. Numbers indicate n and % or median and IQR if indicated in the first column. One participant in the partial or no recovery group had a mild aortic valve regurgitation; no participant had aortic or mitral valve stenosis

### Primary outcome

After a median follow-up of 8.98 months (IQR 5.72–29.37), 76/110 (69.1%, 95% CI 59.6–77.6%) participants had a full recovery (LVEF > 50%), 22/110 (20%) a partial recovery (LVEF of 35 to 50%) of the LV systolic function, and 12/110 (10.9%) remained with a persistent reduced LV systolic function (LVEF of < 35%). The recovery rate of the LV systolic function was similar in the group of participants who had their baseline echocardiography done between 2015 and 2020 (46/68, 67.6%, 95% CI 55.2–78.5%) compared to those who had a baseline echocardiography during the study period from February 2021 to September 2021 (30/42, 71.4%, 95% CI 55.4–84.3%).

**Fig. 2:**
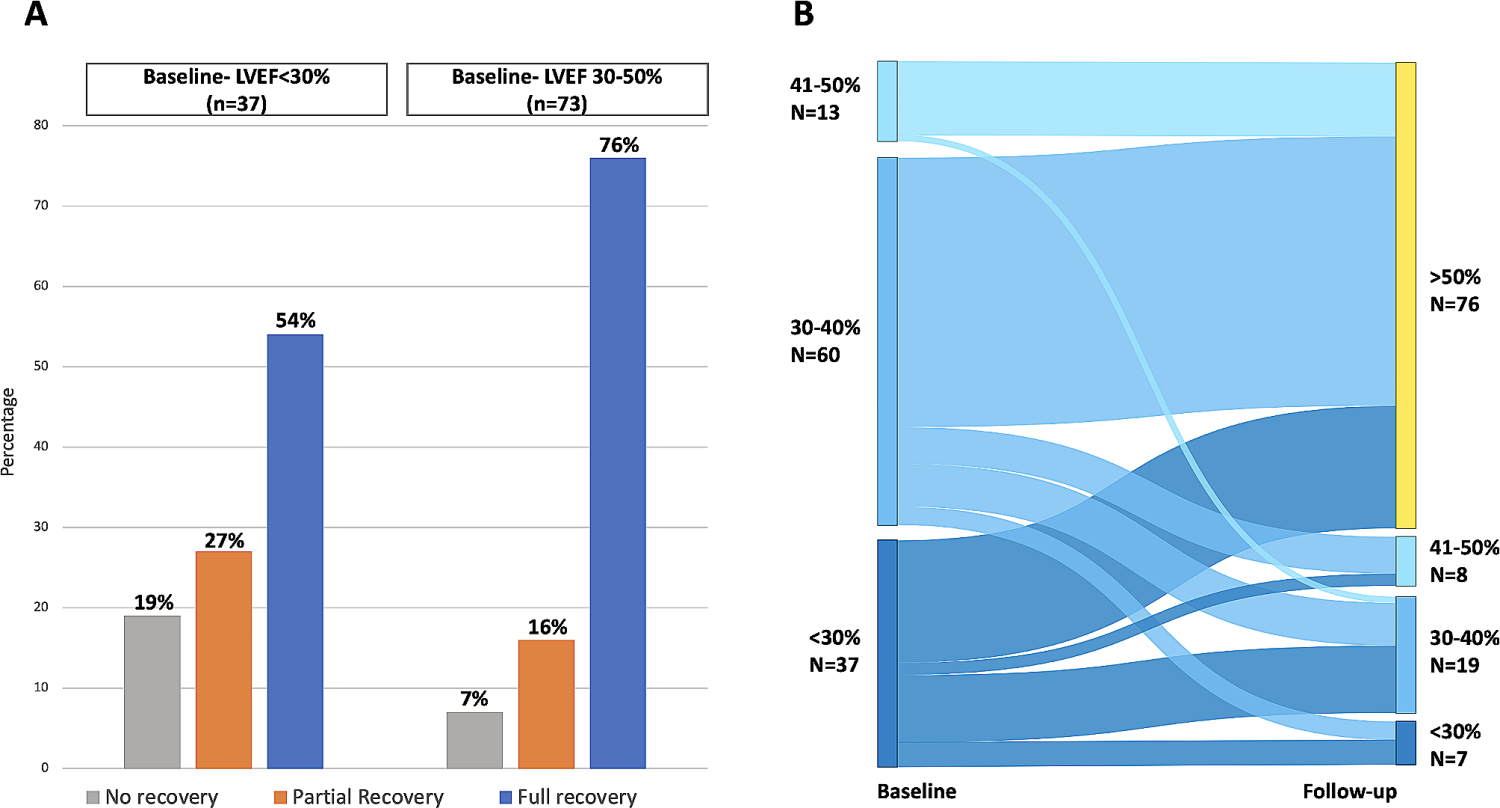
**A**: Outcome of 110 participants according to baseline LVEF (< 30% or ≥ 30%). **B**: Change of LVEF according to baseline LVEF in individual participants LVEF, left ventricular ejection fraction

Figure 2 A shows the primary outcome according to the severity of LV systolic function impairment at baseline. The LV systolic function recovered fully in 54% of those with severe heart failure at baseline and in 76% of those with moderate- or mild heart failure at baseline. Figure 2B shows changes of the LVEF according to baseline LVEF in individual participants, and Figure S1 individual LVEF changes from baseline to follow-up.

### Predictors of full recovery of left ventricular systolic function

In the univariate analysis, LVEF at baseline (for each 5% increase, OR 1.6, 95% CI 1.19–2.15, *p* = 0.002) was significantly associated with recovery of the LV systolic function, while age (for each 10 years older, OR 0.5, 95% CI 0.28-0-92, *p* = 0.026), orthopnea (OR 0.32, 95% CI 0.12–0.88, *p* = 0.027), and palpitations (OR 0.11, 95% CI 0.01–0.98, *p* = 0.048) were inversely associated with full recovery of the LV systolic function. In the multivariate model, LVEF at baseline (for each 5% increase, OR 1.7, 95% CI 1.10–2.62, *p* = 0.012) remained associated with recovery of the LV systolic function. Age (for each 10 years older, OR 0.40, 95% CI 0.19–0.82, *p* = 0.012) remained inversely associated with full recovery of the LV systolic function (Table 2).

**Table 2:** Predictors of full recovery of the left ventricular systolic function (LVEF > 50%) in 110 participants with PPCM

Variable (at baseline)	Univariate analysis			Multivariate analysis*		
	OR	95% CI	*p*-value	OR	95% CI	*p*-value
Age, for each 10 years older	0.50	0.28–0.92	**0.026**	0.40	0.19–0.82	**0.012**
BMI, for each 5 kg/m^2^ increase	0.78	0.48–1.27	0.315	1.09	0.54–2.20	0.803
Arterial hypertension	0.43	0.18–1.04	0.062	0.61	0.19–1.94	0.401
Intake of any cardiac medication	0.57	0.24–1.35	0.203	0.55	0.18–1.69	0.297
Orthopnoea	0.32	0.12–0.88	**0.027**	0.42	0.11–1.63	0.211
Dyspnoea	0.50	0.15–1.63	0.250	1.53	0.29–8.10	0.618
Palpitations	0.11	0.01–0.98	**0.048**	0.27	0.02–4.69	0.370
LVEF at baseline, for each 5% increase	1.60	1.19–2.15	**0.002**	1.70	1.10–2.62	**0.012**
LVEDD	0.96	0.90–1.03	0.204	1.01	0.91–1.11	0.889
LVIDS	0.86	0.91–1.16	0.160	0.95	0.82–1.09	0.470
LV mass index	0.99	0.99–1.01	0.970			
LAVI	0.99	0.97–1.12	0.635			
Diastolic relaxation impairment grade 3	0.39	0.15–1.03	0.057	0.49	0.13–1.72	0.258
Pulmonary arterial hypertension	0.96	0.93–1.01	0.117			
Mitral valve regurgitation	0.30	0.06–1.42	0.131			
Time of follow-up (for each month longer)	1.02	0.99–1.04	0.193	1.03	0.99–1.07	0.147

*Adjusted for all variables listed

OR, odds ratio; CI, confidence interval; LVEF, left ventricular ejection fraction; LVEDD, left ventricular enddiastolic diameter; LVIDS, left ventricular internal diameter end systole; LV mass index, left ventricular mass index; LAVI, left atrial volume index

### Secondary outcomes

At the follow-up visit, a total of 61 (55%) of the participants were still adhering to at least one cardiac medication. Of these, 49 (45%) took a betablocker, 50 (45%) an ACE-inhibitor, 38 (35%) furosemide, and 18 (17%) spironolactone (Table S4). In total, 9 participants died, 3 from the group of participants who had the baseline echocardiogram during the study period in 2021. Cause of death was heart failure in all 3 participants, the cause of death of the others remained unknown.

In the analysis of the outcome of newborns, there was no difference in gestational age at birth, mode of delivery, Apgar score, or rate of death of those born by mothers with full recovery of the LV systolic function compared with those of mothers with partial or no recovery of the LV systolic function (Table S5). Overall, there were only 7/105 (7%) children with a low birth weight of < 2500 g. The median birth weight was lower in children of participants with partial or no recovery (median birth weight 3050 mg (IQR 2500–3500 g) vs. 3300 mg (IQR 2950-3800 mg), *p* = 0.053). In total, 8/110 (7%) of the newborns died. Table S6 shows ECG findings: At baseline or during follow-up, all participants had a sinus rhythm; at follow-up visits, 44% of the participants had a negative T wave, and 3 and 1 participant had a complete right bundle branch block and a complete left bundle branch block, respectively.

## Discussion

In this observational study done in rural East Africa, full recovery of the LV systolic function occurred in 69% of the participants with PPCM over a median follow-up duration of 8.98 months. Predictors of full recovery of the LV systolic function were LVEF at baseline and age, with a higher LVEF at baseline being associated with an improved recovery, and an older age being inversely associated with improvement of the LV systolic function.

In studies from South Africa, Nigeria, and the Philippines, recovery rates of the left ventricular systolic function were as low as 21%, 24%, and 39% [6, 7, 19]. Full recovery of the LV systolic function was defined as LVEF ≥ 55% in the two studies from Africa and > 50% in the Philippine study. In our study, a total of 66 (60%) participants with recovery of their LV systolic function had a LVEF of at least 55%. In a recent meta-analysis, recovery occurred in 37.1% (95% CI 24.8–50.3%) at 6 months and 51.5% (95% CI 31.1–71.7%) at 12 months in participants from sub-Saharan Africa [10].

A left ventricular recovery rate (LVEF ≥ 50%) of 72% was recorded at 12 months in the American IPAC study: At follow-up, black women had a significantly lower LVEF, with only 59% achieving a LVEF ≥ 50% compared with 77% in white females [9]. In women diagnosed with PPCM from Germany, full left ventricular recovery rates after 6 months, 1 year and 5 years were 48%, 60% and 72%, respectively [8]. Studies from other high-and middle-income countries such as Japan, China, and Turkey, described left ventricular recovery rates at 6 months of 30–63% [20–22]. In the study from Turkey, 30% recovered within 6 months, while 70% recovered within 30 months [22]. The higher proportion of participants with a recovered LV systolic function in our study compared to the other two studies from sub-Saharan Africa [6, 7] might be explained by the following: First, while age and the baseline LV function was similar in all three studies, the time between delivery and enrolment (and initiating heart failure therapy), was about half in our study compared to the Nigerian study (median 3 vs. 7 months) [7]. Shorter time from delivery to study inclusion was associated with LV systolic function recovery in the IPAC study [9]. Second, in our study, all participants were prescribed guideline-directed medical therapy for heart failure at baseline by a physician, were followed in a specialized clinic, and 45% were taking an ACE-inhibitor and a betablocker at the follow-up visit. In contrast, participants in the Nigerian study had poor access to specialized care, and in one third and in one fourth only, respectively, ACE-inhibitors and betablockers were prescribed [7]. In the south African study, 80% received an ACE-inhibitor, and 57% only a betablocker at enrolment [6]. Third, an LVEF of 55% was used to define recovery of the LV function in those studies, while we used an LVEF of 50%. Fourth, we could include only those patients who reached the hospital. This might have led to an under-representation of patients with severe heart disease.

In our study, the LVEF at baseline was the strongest predictor of recovery, which is in line with other studies [3, 9, 10]. While age was associated with poorer outcome in our study, age did not reach significance as a predictor of worse outcome in other studies [10]. The left ventricular diameter was not predictive of outcome in our study. This stands in contrast to other studies in which a smaller LV end-diastolic or end-systolic diameter of the LV at baseline was associated with a favourable outcome [10].

The mortality in the group who had their baseline echocardiography in 2021 was 7%, which is comparable to the 6% mortality in the registry of the European Society of Cardiology Observational Research Programme (ESC EORP registry) [11].

In our study, newborns of mothers with PPCM had a median birth weight exceeding 3000 g. Notably, only 7% of these newborns had a low birth weight less than 2500 g. This outcome is more favorable compared to data reported from the ESC EORP registry, which reported a mean birth weight below 3000 g, and up to one third of newborns had low birth weight [11]. The difference in our study might be explained by a different comorbidity profile of our study participants, and by a certain selection bias. Neonatal mortality was 7%, consistent with the ESC EORP registry.

Our study has strengths: To date, this is the first study to include study participants with PPCM from a rural area in sub-Saharan Africa who received specialized care. All echocardiograms were done according to international guidelines by experienced echocardiographers.

Our study has limitations: First, 63 patients were not available for a follow-up examination despite physical tracking. However, the primary outcome did not differ between the retrospectively and the prospectively enrolled cohort. Second, we included participants who came to the clinic. Patients with severe disease may have died or be unable to return for follow-up. Therefore, these patients may be underrepresented. This might have resulted in selection bias and should be considered in the interpretation of results. However, this represents a real-life patient population seen at a referral hospital. Third, drugs had to be paid for by the participants or their relatives. This might be the main reason why only 55% of the patients were taking any cardiac medication at follow-up, even though patients were counseled not to stop taking medication. For those who were not taking medication at follow-up, we were not able to reliably determine the duration of drug intake. Fourth, due to the small number of deaths, we could not define any risk factors for death. Fifth, we could not exclude that arterial hypertension was the main cause of heart disease in hypertensive participants. However, arterial hypertension and hypertensive disorders during pregnancy are risk factors for and common in patients with PPCM [1–3]: Hypertension has been reported to be present in up to 20 to 45% of patients with PPCM [7–9]. Last, this was a single-center study and our results might not be generalizable.

In conclusion, the LV systolic function recovered completely in 69% of participants with PPCM from rural Tanzania who received specialized care, with a median follow-up of 8.98 months. LVEF and age at baseline were the strongest predictors of recovery in this study. Women with PPCM need specialized care for the diagnosis and treatment of their condition, along with clinical and echocardiographic follow-up to adjust and maximise adherence to treatment.

### Electronic supplementary material

Below is the link to the electronic supplementary material.

Supplementary Material 1

## Data Availability

The datasets used and/or analysed during the current study is available from the corresponding author on reasonable request.

## Abbreviations

ACE: angiotensin converting enzyme
BNP: brain natriuretic peptide
ECG: electrocardiogram
LVEF: left ventricular ejection fraction
LV: left ventricle
PPCM: peripartum cardiomyopathy
