# Supplementary material for: Recovery of left ventricular systolic function in peripartum cardiomyopathy: an observational study from rural Tanzania
Source: BMC Cardiovasc Disord. 2024 May 9;24:243. doi: 10.1186/s12872-024-03906-y (PMC11080156; doi:10.1186/s12872-024-03906-y)
Supplement: Supplementary file 1 — Supplementary Material 1 [file 12872_2024_3906_MOESM1_ESM.docx]

**Table S1. Detailed echocardiographic findings of included study participants (n=110)**

|  | **Baseline** | | **Follow-up** | |
| --- | --- | --- | --- | --- |
|  | **n** | **Median (range)/**  **n (%)** | **n** | **Median (range)/**  **n (%)** |
| **Dimensions and LV function** | | | | |
| LVEDD [mm], median (range) | 110 | 57.1 (39-79) | 108 | 47.4 (39.6-81.6) |
| IVSd [mm], median (range) | 110 | 8.05 (4.3-15.5) | 108 | 7.4 (4.9-13) |
| PWd [mm], median (range) | 97 | 9 (5.3-12.9) | 107 | 7.15 (4.2-16) |
| LVIDs [mm], median (range) | 97 | 50 (10-74) | 107 | 41.34 (6-68) |
| LVOT [mm], median (range) | 109 | 17.7 (13-23) | 108 | 19 (12-25) |
| Aorta asc [mm], median (range) | 107 | 25 (14.2-41) | 108 | 29 (20-38) |
| LA [mm], median (range) | 109 | 40 (21-70) | 103 | 30 (20-58) |
| LA [ml], median (range) | 109 | 67 (18-179) | 105 | 34 (12-182) |
| LAVI [ml/m^2^], median (range) | 106 | 40.5 (10-98) | 100 | 22 (8-133) |
| RA [mm], median (range) | 4 | 40 (17-83) | 13 | 26 (18-49) |
| RA [ml], median (range) | 105 | 48 (14-178) | 96 | 24 (10-109) |
| LV mass Index [g/m^2^], median (range) | 107 | 118 (11-246) | 104 | 75 (41-213) |
| LV mass [grams], median (range) | 80 | 192 (118-406) | 104 | 116.5 (67-346) |
| RWT median (range) | 80 | 0.32 (0.15-0.47) | 104 | 0.3 (0.18-0.58) |
| V. cava [mm], median (range) | 107 | 16.7 (1.6-33) | 106 | 12 (5-26) |
| V. cava collapsing >50% during inspiration, n (%) | 108 | 68 (63.0) | 109 | 97 (89.0) |
| V. cava insp. [mm], median (range) | 105 | 2.2 (0-24) | 105 | 2.2 (1-24) |
| Pericardial effusion, n (%) | 102 | 23 (22.5%) | 106 | 7 (6.6) |
| Pericardial effusion [mm], median (range) | 23 | 5 (1-17.6) | 7 | 1 (1-12.6) |
| EF [%], median (range) | 110 | 30 (15-46) | 110 | 55.5 (15-68) |
| EF >50%, n (%) | 110 | 0 (0.0) | 110 | 76 (69.1) |
| EF 41%-50%, n (%) | 110 | 13 (11.8) | 110 | 8 (7.3) |
| EF 30%-40%, n (%) | 110 | 60 (54.5) | 110 | 19 (17.3) |
| EF <30%, n (%) | 110 | 37 (33.6) | 110 | 7 (6.4) |
| Regional wall motion, normal, n (%) | 110 | 15 (13.6) | 108 | 78 (72.2) |
| Regional wall motion, hypo-akinnesia, n (%) | 110 | 94 (85.5) | 108 | 29 (26.9) |
| Regional wall motion, hyperdynamic, n (%) | 110 | 0 (0.0) | 108 | 0 (0.0) |
| Regional wall motion, d-sign, n (%) | 110 | 1 (0.9) | 108 | 1 (0.9) |
| **Right ventricle** | | | | |
| RV mid [mm], median (range) | 101 | 29.7 (13-54) | 109 | 26 (10-44) |
| TAPSE [mm], median (range) | 110 | 18.25 (8-35) | 108 | 21 (9-34) |
| Sa [cm/s], median (range) | 106 | 10 (5.68-17) | 103 | 12 (7.78-21) |
| RV basal [mm], median (range) | 110 | 40.5 (24-66) | 109 | 34 (11-58.6) |
| FAC [%], median (range) | 109 | 20 (10-45) | 19 | 31 (10-40) |
| MPI, median (range) | 13 | 0.82 (0.46-3) | 19 | 0.6 (0.3-1.53) |
| PVAccT/RVET, median (range) | 4 | 0.755 (0.5-60) | 1 | 63 (63-63) |
| **Diastolic function** | | | | |
| E, median (range) | 109 | 103 (45-162) | 110 | 80.5 (37-164) |
| A, median (range) | 108 | 40 (10-113) | 110 | 60 (19-119) |
| E/A, median (range) | 108 | 2.285 (0.5-6.29) | 110 | 1.34 (0.5-5.73) |
| septal e`, median (range) | 107 | 6.12 (2.5-14) | 110 | 8 (4.42-15) |
| lateral e`, median (range) | 108 | 7.895 (3.7-14) | 109 | 10 (3.33-20) |
| E/e`, median (range) | 107 | 15.3 (3.21-29) | 109 | 10 (4.12-33) |
| DT [ms], median (range) | 108 | 108 (6-294) | 109 | 146 (15-276) |
| Ar-A >30ms, n (%) | 108 | 7 (7.6) | 102 | 1 (1.0) |
| PveinS/D, median (range) | 92 | 1 (0.23-2.76) | 95 | 1.2 (0.48-2) |
| Pulmonary arterial pressure [mmHg], median (range) | 92 | 40 (12-63) | 53 | 27 (10-58) |
| **Valves** | | | | |
| Aortic valve regurgitation, none, n (%) | 110 | 90 (81.8) | 108 | 103 (95.4) |
| Aortic valve regurgitation, minimal, n (%) | 110 | 13 (11.8) | 108 | 3 (2.8) |
| Aortic valve regurgitation, mild, n (%) | 110 | 6 (5.5) | 108 | 1 (0.9) |
| Aortic valve regurgitation, mild-moderate, n (%) | 110 | 1 (0.9) | 108 | 1 (0.9) |
| Aortic valve stenosis, none, n (%) | 110 | 110 (100.0) | 108 | 108 (100.0) |
| Aortic valve cusps, normal, n (%) | 110 | 108 (100.0) | 108 | 108 (100.0) |
| PG mean aortic [mmHg], median (range) | 106 | 2 (0.49-7.71) | 101 | 2.5 (0.6-7) |
| VC aortic [mm], median (range) | 11 | 1.9 (0-3.2) | 2 | 9.3 (6.1-12.5) |
| PHT aortic [ms], median (range) | 6 | 333 (0-420) | 2 | 306 (300-312) |
| Vmax aortic [cm/s], median (range) | 7 | 232 (0-265) | 9 | 110 (56-894) |
| Mitral valve stenosis, none, n (%) | 109 | 109 (100.0) | 107 | 107 (100.0) |
| Mitral valve leaflets, normal, n (%) | 104 | 103 (99.0) | 106 | 106 (100.0) |
| Mitral valve leaflets, sclerosis, n (%) | 104 | 1 (1.0) | 106 | 0 (0.0) |
| Mitral valve regurgitation, none, n (%) | 110 | 5 (4.5) | 109 | 66 (60.6) |
| Mitral valve regurgitation, minimal, n (%) | 110 | 10 (9.1) | 109 | 11 (10.1) |
| Mitral valve regurgitation, mild, n (%) | 110 | 78 (70.9) | 109 | 27 (24.8) |
| Mitral valve regurgitation, mild-moderate, n (%) | 110 | 11 (10.0) | 109 | 2 (1.8) |
| Mitral valve regurgitation, moderate, n (%) | 110 | 5 (4.5) | 109 | 1 (0.9) |
| Mitral valve regurgitation, moderate-severe, n (%) | 110 | 0 (0.0) | 109 | 1 (0.9) |
| Mitral valve regurgitation, severe, n (%) | 110 | 1 (0.9) | 109 | 1 (0.9) |
| r mitral [mm], median (range) | 110 | 5.2 (0-10.2) | 34 | 4.65 (0-7.9) |
| Vr mitral, median (range) | 95 | 22 (20-42) | 28 | 22 (22-95) |
| Vmax mitral, median (range) | 91 | 388 (263-543) | 30 | 369 (232-532) |
| VTI mitral, median (range) | 91 | 113 (54-286) | 30 | 112 (51-186) |
| EROA mitral, median (range) | 91 | 10 (2-48) | 30 | 11 (3-42) |
| Rvol mitral, median (range) | 90 | 11 (2-48) | 27 | 12 (3-53) |
| Tricuspid valve regurgitation, none, n (%) | 108 | 22 (20.4) | 106 | 74 (69.8) |
| Tricuspid valve regurgitation, minimal, n (%) | 108 | 30 (27.8) | 106 | 21 (19.8) |
| Tricuspid valve regurgitation, mild, n (%) | 108 | 28 (25.9) | 106 | 3 (2.8) |
| Tricuspid valve regurgitation, mild-moderate, n (%) | 108 | 4 (3.7) | 106 | 3 (2.8) |
| Tricuspid valve regurgitation, moderate, n (%) | 108 | 11 (10.2) | 106 | 2 (1.9) |
| Tricuspid valve regurgitation, moderate-severe, n (%) | 108 | 6 (5.6) | 106 | 1 (0.9) |
| Tricuspid valve regurgitation, severe, n (%) | 108 | 7 (6.5) | 106 | 2 (1.9) |
| PG max tricuspid [mmHg], median (range) | 95 | 30 (0-47) | 58 | 16 (0-43) |
| TR velocity tricuspid >2.8m/s, n (%) | 95 | 46 (48.4) | 53 | 43 (81.1) |
| Pulmonal valve regurgitation, none, n (%) | 109 | 66 (60.6) | 107 | 105 (98.1) |
| Pulmonal valve regurgitation, minimal, n (%) | 109 | 25 (22.9) | 107 | 1 (0.9) |
| Pulmonal valve regurgitation, mild, n (%) | 109 | 18 (16.5) | 107 | 0 (0.0) |
| Pulmonal valve regurgitation, moderate-severe, n (%) | 109 | 0 (0.0) | 107 | 1 (0.9) |
| Pulmonal valve stenosis, none, n (%) | 109 | 109 (100.0) | 107 | 107 (100.0) |
| PVAccT pulmonal [m/s], median (range) | 55 | 90 (36-162) | 98 | 123 (38-200) |
| **Interpretation of echocardiographic findings** | | | | |
| Normal LV size, n (%) | 110 | 9 (8.2) | 110 | 81 (73.6) |
| Eccentric hypertrophy of LV, n (%) | 101 | 99 (98.0) | 29 | 28 (96.6) |
| Concentric hypertrophy, n (%) | 101 | 1 (1.0) | 29 | 1 (3.4) |
| Concentric remodelling, n (%) | 101 | 1 (1.0) | 29 | 0 (0.0) |
| Eccentric hypertrophy LV, none, n (%) | 108 | 9 (8.3) | 109 | 81 (74.3) |
| Eccentric hypertrophy LV, severe, n (%) | 108 | 50 (46.3) | 109 | 16 (14.7) |
| Eccentric hypertrophy LV, moderate, n (%) | 108 | 23 (21.3) | 109 | 7 (6.4) |
| Eccentric hypertrophy LV, mild, n (%) | 108 | 26 (24.1) | 109 | 5 (4.6) |
| Diastolic relaxation impairment, none, n (%) | 107 | 2 (1.9) | 110 | 77 (70.0) |
| Diastolic relaxation impairment, grade 1, n (%) | 107 | 22 (20.6) | 110 | 6 (5.5) |
| Diastolic relaxation impairment, grade 2, n (%) | 107 | 13 (12.1) | 110 | 6 (5.5) |
| Diastolic relaxation impairment, grade 3, n (%) | 107 | 70 (65.4) | 110 | 21 (19.1) |
| Elevated LV-filling pressure, n (%) | 107 | 83 (77.6) | 110 | 26 (23.6) |
| Right ventricle size dilated, n (%) | 109 | 48 (44.0) | 110 | 17 (15.5) |
| Right ventricle function impaired, n (%) | 109 | 55 (50.5) | 110 | 14 (12.7) |
| Pulmonary hypertension (>40mmHg), none, n (%) | 103 | 54 (52.4) | 110 | 97 (88.2) |
| Pulmonary hypertension (>40mmHg), caused by left sided heart disease (i.e. elevated LV filling pressure), n (%) | 103 | 49 (47.6) | 110 | 13 (11.8) |
| LV apical thrombus, n (%) | 110 | 4 (3.6) | 110 | 0 (0.0) |

*LVEDD, Left ventricular enddistolic diameter; IVSd, inraventricular septum in diastole; PWd, posterior wall in diastole; LVIDs, left ventricular internal diameter end systole; LVOT, left ventricular outflow tract; LA, left atrium; LAVI, left atrial volume index; RA, right atrium; LV, left ventricular; RWT, relative wall thickness; EF, left ventricular ejection fraction; RV, right ventricular; TAPSE, tricuspid annular plane systolic excursion; FAC, fractional area change; MPI, myocardial performance index; PV Acct, pulmonary valve acceleration time; RVET, right ventricular ejection time; PveinS/D, pulmonary vein velocity systolic/diastolic; PG mean, mean pressure gradient; PHT, pressure half time; Vmax, maximal velocity; VC, vena contracta; VTI, velocity time integral; EROA, effective regurgitant orifice area; Rvol, regurgitant volume*

**Table S2. Additional data of the prospective cohort (n=42)**

| **Variable (at baseline)** | **Full recovery  (EF >50%) (n=30)** | | **Partial or no recovery (n=12)** | | **p-value** |
| --- | --- | --- | --- | --- | --- |
|  | **n** | **%** | **n** | **%** |  |
| Current breastfeeding | 29 | 96.7 | 7 | 58.3 | **0.001** |
| Parity, IQR | 2 | 2-4 | 3 | 2-4 | 0.859 |
| Fatigue | 11 | 36.7 | 10 | 83.3 | **0.006** |
| Limb swelling | 20 | 66.7 | 6 | 50 | 0.315 |
| Abdominal swelling | 3 | 10 | 7 | 58.3 | **0.001** |
| Nocturia | 1 | 3.3 | 1 | 8.3 | 0.495 |
| Dizziness | 3 | 10 | 0 | - | - |
| Palpitations | 20 | 66.7 | 11 | 91.7 | 0.096 |
| Gestational arterial hypertension | 1 | 3.3 | 2 | 16.7 | 0.192 |
| HELLP syndrome | 1 | 3.3 | 0 | - | - |
| Median hemoglobin (g/dl), IQR | 13 | 12-14 | 13.6 | 12-15 | 0.498 |
| Median creatinine (mmol/L), IQR | 62 | 51-74 | 68 | 66-95 | 0.125 |
| Median glucose (mg/dl), IQR | 88 | 70-96 | 94 | 92-112 | 0.098 |
| Median SpO2, IQR | 98 | 96-98 | 98 | 96-99 | 0.820 |
| Median BNP (pg/ml), IQR | 250 | 120-877 | 2030 | 90-4475 | 0.147 |
| Repolarization abnormality in ECG | 15 | 50 | 10 | 83.3 | **0.048** |

*IQR, interquartile range; BNP, brain natriuretic peptide; ECG, electrocardiogram; Repolarization abnormality: Negative T wave or flat T wave in more than 2 leads in a 12 channel ECG. No participant had a syncope, no participant had cyanosis.*

**Table S3. Comparison of baseline parameters (excluded vs included participants)**

| **Baseline parameters** | **Excluded (n=92)** | **Included (n=110)** | **p-value** |
| --- | --- | --- | --- |
| **Sociodemographic characteristics** | | | |
| Age (n=92)/(n=110), median (range) | 30 (16-46) | 28.5 (17-45) | 0.714 |
| BMI (n=91)/(n=109), median (range) | 23.2 (17.6-35.2) | 22.6 (14.8-36.9) | 0.784 |
| Inpatient at baseline (n=78)/(n=110), n (%) | 36 (46.2) | 35 (31.8) | **0.046** |
| Profession, farmer (n=58)/(n=110), n (%) | 53 (91.4) | 90 (81.8) | 0.098 |
| **Clinical parameters** | | | |
| Arterial hypertension (n=92)/(n=110), n (%) | 34 (36.9) | 24 (21.8) | **0.018** |
| **Dimensions and LV function** | | | |
| LVEF [%] (n=92)/(n=110), median (range) | 30 (15-50) | 30 (15-46) | 0.091 |
| LVEF 41%-50% (n=92)/(n=110), n (%) | 4 (4.3) | 13 (11.8) | 0.075 |
| LVEF 30%-40% (n=92)/(n=110), n (%) | 52 (56.5) | 60 (54.5) | 0.887 |
| LVEF <30% (n=92)/(n=110), n (%) | 36 (39.1) | 37 (33.6) | 0.463 |
| LVEDD (n=92)/(n=110), median (range) | 57.5 (44-74) | 57.1 (39-79) | 0.915 |
| LV mass Index [g/m^2^] (n=88)/(n=107), median (range) | 130 (47-248) | 118 (11-246) | **0.044** |
| LAVI [ml/m^2^] (n=91)/(n=106), median (range) | 43 (10-86) | 40.5 (10-98) | 0.880 |
| **Interpretation of echocardiographic findings** | | | |
| Eccentric hypertrophy of LV, severe (n=76)/(n=108), n (%) | 38 (50) | 50 (46.3) | 0.620 |
| Diastolic relaxation impairment, grade 3 (n=92)/(n=107), n (%) | 53 (57.6) | 70 (65.4) | 0.246 |

*BMI, body mass index; LVEF, left ventricular ejection fraction; LVEDD, left ventricular enddiastolic diameter; LAVI, left atrial volume index; LV, left ventricle*

**Table S4. Medication intake at follow-up**

| **Medication** | **Participants, n (%)** |
| --- | --- |
| **Diuretic** | |
| Furosemide | 38 (34.9) |
| **Mineralocorticoid receptor antagonist** | |
| Spironolactone | 18 (16.5) |
| **Beta-blockers** | |
| Carvedilol | 47 (43.1) |
| Bisoprolol | 2 (1.8) |
| **ACE-inhibitors** | |
| Captopril | 2 (1.8) |
| Lisinopril | 12 (11.0) |
| Enalapril | 36 (33.0) |
| **Others** | |
| Bromocriptine | 0 (0.0) |
| Calcium channel blockers | 0 (0.0) |
| Digoxin | 8 (7.3) |

**Table S5. Characteristics of children according to maternal recovery of the left ventricular systolic function**

| **Variable** | **Full recovery**  **(EF >50%)**  **(n=76)** | | **Partial or no recovery**  **(n=34)** | | **p-value** |
| --- | --- | --- | --- | --- | --- |
|  | **n** | **% or IQR** | **n** | **% or IQR** |  |
| Median gestational age at birth, IQR | 40 | 40-40 | 40 | 39-40 | 0.825 |
| Cesarean mode of delivery* | 15/73 | 20.6 | 10/33 | 31.3 | 0.236 |
| Apgar score at 5 minutes of 10** | 40/40 | 100 | 17/19 | 89.5 | 0.100 |
| Median birth weight***, IQR | 3300 | 2950-3800 | 3050 | 2500-3500 | **0.053** |
| Child cried right after birth**** | 69/71 | 97.2 | 27/30 | 90 | 0.907 |
| Baby died | 5/76 | 7.0 | 3/34 | 10.0 | 0.442 |

*IQR, interquartile range; * Information missing for 4 patients; ** information missing for 51 children; *** information missing for 5 children; **** information missing for 9 children*

**Table S6. ECG findings**

|  | **Baseline prospective cohort (n=42)** | **Follow-up* prospective cohort (n=41)** | **Follow-up retrospective cohort (n=68)** | **Follow-up* overall (n=109)** |
| --- | --- | --- | --- | --- |
| **Rythm** | | | | |
| Sinus rhythm, n (%) | 42 (100) | 41 (100) | 68 (100) | 109 (100) |
| Sinus tachycardia, n (%) | 24 (57.1) | 4 (9.8) | 10 (14.7) | 14 (12.8) |
| Sinus bradycardia, n (%) | 3 (7.1) | 10 (24.4) | 2 (2.9) | 12 (11.0) |
| Bigeminus, n (%) | 2 (4.8) | 0 (0) | 1 (1.5) | 1 (0.9) |
| Ventricular extra beats, n (%) | 2 (4.8) | 2 (4.9) | 2 (2.9) | 2 (1.8) |
| **Conduction abnormalities** | | | | |
| Right bundle branch block, n (%) | 1 (2.4) | 2 (4.9) | 1 (1.5) | 3 (2.8) |
| Left bundle branch block n (%) | 2 (4.8) | 0 (0.0) | 1 (1.5) | 1 (0.9) |
| **Repolarization** | | | | |
| Normal, n (%) | 17 (40.5) | 23 (56.1) | 28 (41.2) | 51 (46.8) |
| Negative T wave, n (%) | 21 (50.0) | 14 (34.1) | 34 (50.0) | 48 (44.0) |
| Other repolarization abnormalities, n (%) | 4 (9.5) | 4 (9.8) | 6 (8.8) | 10 (9.2) |
| **Sign of LV hypertrophy** | | | | |
| Sokolov index > 3.5, n (%) | 11 (26.2) | 3 (7.3) | 30 (44.1) | 33 (30.3) |

*ECG data at baseline was only collected for the prospective cohort (n=42). * ECG data missing in 1 participant; tachycardia, heart rate >100 beats per minute; bradycardia, heart rate <60 beats per minute. Heart rate of bradycardic participants ranged between 47 and 59 beats per minute. LV, left ventricular.*

**Figure S1. Individual LVEF change of 110 participants from baseline to follow-up**


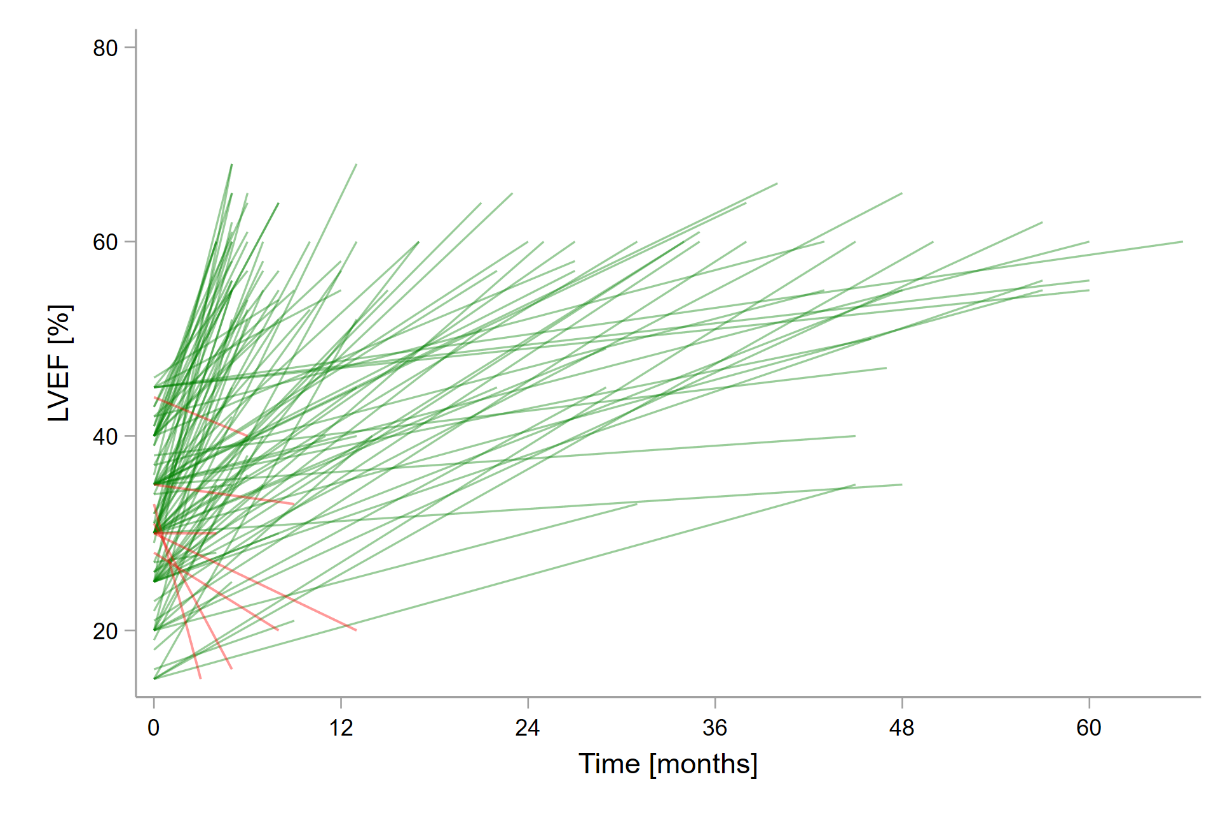


*LVEF, left ventricular ejection fraction*

**Comment on Figure S1:** Changes from baseline to follow-up is shown in green for participants with increasing LVEF and in red for participants with decreasing LVEF. Time points of follow-up variations ranged from 3.32 months to 66.27 months.
